# Does Surgical Repair Benefit Pipkin Type I Femoral Head Fractures?: A Systematic Review and Meta-Analysis

**DOI:** 10.3390/life12010071

**Published:** 2022-01-05

**Authors:** Sung Huang Laurent Tsai, Wei-Che Tai, Tsai-Sheng Fu, Eric H. Tischler, Rafa Rahman, Yong-Kuan Lim, Yi-Hsun Yu, Chun-Yi Su

**Affiliations:** 1 Department of Orthopaedic Surgery, Chang Gung Memorial Hospital, Keelung branch, Keelung 204 and School of Medicine, Chang Gung University, Taoyuan 333, Taiwan; stsai29@jh.edu (S.H.L.T.); fts111@cgmh.org.tw (T.-S.F.); jaykuan86@cgmh.org.tw (Y.-K.L.); 2 The Johns Hopkins Bloomberg School of Public Health, Johns Hopkins University, Baltimore, MD 21205, USA; etischle@nyit.edu (E.H.T.); rafarahman@jh.edu (R.R.); 3 Department of Orthopaedic Surgery, Chang Gung Memorial Hospital, Linkou branch, and School of Medicine, Chang Gung University, Taoyuan 333, Taiwan; docfred@cgmh.org.tw (W.-C.T.); m7048@adm.cgmh.org.tw (Y.-H.Y.); 4 Department of Orthopaedic Surgery and Rehabilitation Medicine, Downstate Medical Center, State University of New York, Brooklyn, NY 11203, USA

**Keywords:** femoral fractures, hip dislocation, fracture fixation, arthritis, femur head necrosis

## Abstract

**Background**: Femoral head fractures are rare injuries with or without traumatic dislocations. The management of these fractures is crucial to prevent the development of severe complications and to achieve optimal functional outcomes. Wide treatment options for Pipkin 1 femoral head fractures range from fragment excision, fixation following open reduction with internal fixation, or conservative treatment such as close reduction alone after fracture dislocation. However, the best decision making remains controversial not only due to lack of large trials, but also inconsistent results reported. Therefore, we aim to compare the operative with nonoperative outcomes of Pipkin type 1 patients. **Patients and Methods**: We systemically searched MEDLINE, EMBASE, Cochrane library, In-Process & Other Non-Indexed Citations to identify studies assessing outcomes of Pipkin type 1 patients after conservative treatment, and open reduction with excision or fixation. Data on comparison of clinical outcomes of each management were extracted including arthritis, heterotopic ossification (HO), avascular necrosis (AVN), and functional scores (Thompson Epstein, Merle’ d Augine and Postel Score). We performed a meta-analysis with the available data. **Results**: Eight studies (7 case series and 1 RCT) were included in this study. In a pooled analysis, the overall rate of arthritis was 37% (95% CI, 2–79%), HO was 20% (95% CI, 2–45%), and AVN was 3% (95% CI, 0–16%). In comparison of management types, the excision group reached the best functional outcomes including Thompson Epstein Score (poor to worse, 9%; 95% CI, 0–27%) and Merle d’ Aubigne and Postel Score (poor to worse, 18%; 95% CI, 3–38%); ORIF group had the highest AVN rate (11%; 95% CI, 0–92%); conservative treatment had the highest arthritis rate (67%; 95% CI: 0–100%) and lowest HO rate (2%; 95% CI, 0–28%). **Discussion**: This meta-analysis demonstrates that different procedures lead to various clinical outcomes: fragment excision may achieve better function, conservative treatment may result in a higher arthritis rate, while ORIFs may have a higher AVN rate. These findings may assist surgeons in tailoring their decision-making to specific patient profiles. Future RCTs with multicenter efforts are needed to validate associations found in this study. Level of Evidence: II, systematic review and meta-analysis.

## 1. Introduction

Femoral head fractures are relatively uncommon injuries, with a reported 2 cases per million people per year [1]. Femoral head fractures typically result from high-energy trauma causing compression along the axis of the femur with transmission into the hip joint [2]. Approximately 84% of femoral head fractures result from a high-energy motor vehicle dashboard injury [3,4,5]. Simultaneous hip dislocation [6,7,8,9,10] accounts for 4–17%. To classify and guide definitive treatment of femoral head fractures, the Pipkin classification is most commonly referenced [11,12]. The Pipkin femoral head fracture classification is as following: Type I- fracture is inferior to the fovea capitis femoris, the non-weight bearing surface of the femoral head; Type II- fracture is superior to the fovea capitis femoris, the weight bearing surface of the femoral head; Type III- Either Type I or II with associated femoral neck fracture; Type IV- Either Type I or II with associated acetabular fracture. Treatment options for Pipkin Type I and II injuries include non-operative management if closed reduction achieves <1–2mm displacement and an anatomic congruent hip joint without evidence of fragment interposition [11]. Surgical treatment options include fragment excision or open reduction internal fixation. Of note, Type I and II fractures are associated with better outcomes when compared to Type III and IV fracture patterns [11]. Giannoudis PV et al. conducted a systematic review and reported that 21% of Pipkin type 1 fractures are treated nonoperatively [3]. Pipkin et al. preferred closed reduction alone for these injuries [11]. However, surgery may be indicated if a comminuted fracture fragment prevented successful reduction of the hip joint. Complications, including heterotopic ossification (HO), avascular necrosis (AVN), and post-traumatic arthritis can limit patient’s hip function and postoperative outcomes [6,13,14,15,16,17].

Current evidence is limited on reporting femoral head fracture outcomes according to classification, especially the outcomes in early stage. Guo et al. conducted a systematic review of 10 studies of 176 patients assessing the surgical approach on postoperative femoral head fractures and the development of HO and AVN, revealing that the use of the anterior approach results in a higher risk of HO, while the posterior approach may increase AVN risk [16]. Masse et al. reported that among 13 femoral head fracture patients treated surgically, one patient developed AVN, one developed post-traumatic arthritis, and two developed HO [18]. Furthermore, Lin et al. evaluated mid and long term results of trochanteric flip osteotomy of 9 type I and 14 type II Pipkin femoral head fractures patients. HO, AVN, and post-traumatic arthritis developed in 3,2, and 1 patients, respectively [19].

The optimal treatment for Pipkin type 1 femoral head fractures remains controversial due to mixed results. Therefore, our aim of this study was to perform a systematic review and meta-analysis to compare the operative to nonoperative outcomes of Pipkin type 1 femoral head fractures patients.

## 2. Material and Methods

### 2.1. Study Registration and Guidelines

This systematic review was performed in accordance with the Preferred Reporting Items for Systematic Review and Meta Analyses (PRISMA) statement. Additionally, it was recorded on the PROSPERO database (CRD42020176182).

### 2.2. Literature Search

A comprehensive electronic search of MEDLINE, EMBASE, Scopus, Ovid Cochrane Central Register of Controlled Trials, Ovid Cochrane Database of Systematic Reviews and Web of Science was completed on 5 April 2020. The search strategy was designed to capture all studies that investigated the outcomes of procedures in the context of femoral head infra-foveal type fracture patients. Our PICO approach (patients/population, intervention, comparator, and outcomes) was established in accordance to the following question: For patients with Pipkin 1 femoral head fractures (patients/population), which treatment, including ORIF, fragment excision, or conservative treatment (closed reduction alone) (intervention/comparator) is associated with superior outcomes (outcomes)? Medical Subject Headings (MeSH) were used where appropriate. The search was limited to English language only and excluded animal studies. The full search strategy is available in Table S1.

### 2.3. Independent Review Process

Two independent reviewers screened articles from the literature for inclusion, completed data extraction, and assessed methodological quality. A third author was required to resolve any disagreements.

### 2.4. Study Selection and Eligibility

Eligibility was defined using the Cochrane Collaboration PICOS (population, intervention, comparison, outcome, study design) method [20]. English-language studies were eligible for inclusion if any of the following postoperative follow-up outcomes were included: Thompson Epstein Score, Merle d’Aubigne and Postel Score, arthritis, AVN, and HO (outcome). Treatment intervention options evaluated were closed reduction alone (comparator), open reduction fragment excision, and open reduction internal fragment fixation (interventions) in patients with an infra-foveal type of femoral head fracture (Pipkin type 1) (population). Eligible study designs included randomized controlled trials, cohort studies, and case series – case reports were excluded (study design). All literature results were documented in Covidence.

### 2.5. Data Extraction

Extracted data included study characteristics (study origin, study design, publication date), patient factors (demographics, mechanism of injury), surgical factors (time to surgery, technique), and clinical outcomes (follow-up duration, Thompson Epstein Score, Merle d’Aubigne and Postel Score, post-traumatic arthritis, AVN, HO, functional status, and any complications). The Thompson Epstein and Merle d’Aubigne and Postel Scores have been consistently utilized in the published literature to evaluate pre and postoperative clinical status. Given its simple application and reported reliability, these outcome measurements were included for analysis in this study [21]. For binary outcomes, total number of participants, and number of events were recorded. For continuous outcomes, group size, mean, standard deviations, and adjusted effect estimates and 95% Confidence Intervals (CI) were documented.

### 2.6. Assessment of Methodological Quality and Risk of Bias

The modified Newcastle-Ottawa Scale (NOS, Table 1) was utilized to assess the methodological quality of non-randomized studies in meta-analyses [22]. The Grading of Recommendations Assessment, Development and Evaluation (GRADE) system was used to assess the overall quality of evidence [23].

### 2.7. Outcome Measures, Data Synthesis, and Statistical Analysis

Individual patient data was pooled to form an aggregate dataset. Meta-analysis of pooled data was performed using descriptive statistics and non-parametric statistical tests. Binary outcomes were summarized using a pooled proportion of events (effect size [ES]), with corresponding 95% confidence intervals (CIs) calculated by the Wilson method [28]. Continuous outcomes were pooled using weighted averages (WA) with standard deviations (SDs). Results are graphically represented by forest plots. The Freeman-Tukey transformation was used to stabilize variance to include studies with a zero event rate [29]. DerSimonian and Laird approach random-effects model was used to account for high heterogeneity (>50%) between studies [30]. Between-study heterogeneity was assessed using Cochrane Q and I^2^ statistics. According to the Cochrane handbook, heterogeneity was considered non-important (I^2^ < 30%), moderate (I^2^ 30–60%) and substantial (I^2^ > 60%). Higgens methods were used to assess heterogeneity effect estimate within studies [31]. A *p*-value of <0.05 was considered statistically significant. Publication bias was evaluated by generating funnel plots and examining them for any obvious visual asymmetry [32]. Statistical analysis was conducted using the Stata software (StataCorp. 2019. Stata Statistical Software: Release 16.0 [StataCorp LLP, College Station, TX, USA]).

## 3. Results

### 3.1. Study Selection, Quality Assessment, and Study Characteristics

**Figure 1:**
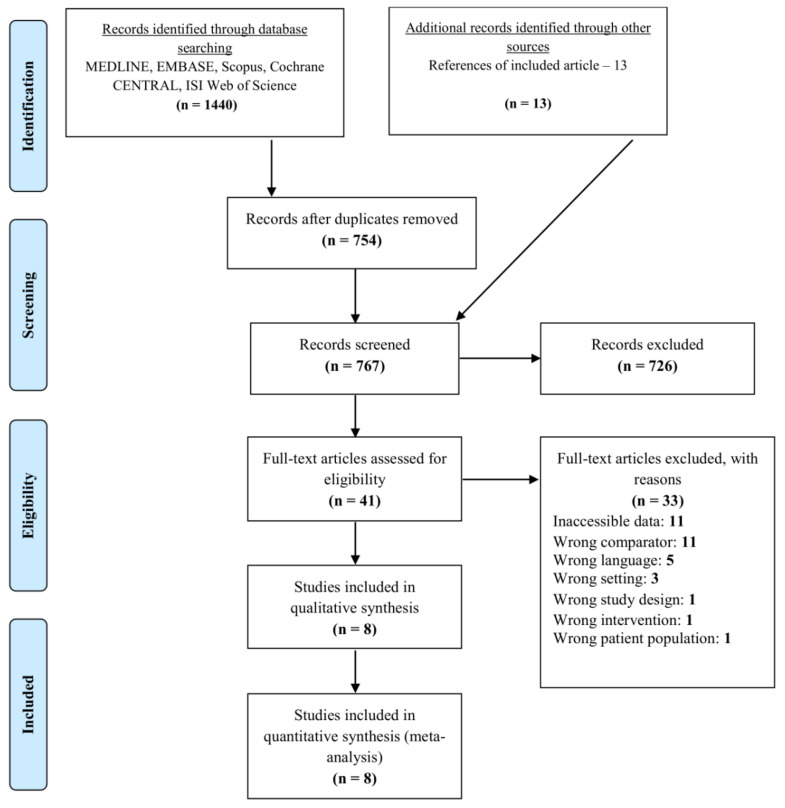
PRISMA flow diagram showing the literature review, search strategy, and selection process for including studies for systematic review and meta-analysis. Flow of identification, screening, eligibility and inclusion.

The PRISMA flow diagram depicts the inclusion qualitative and quantitative criteria for selected articles (Figure 1). The systematic literature search identified 792 articles and 13 additional relevant articles were identified after reviewing the references of the articles generated from the literature search. After removal of duplicates, 767 unique articles remained. Following initial screening, 41 full-text articles were reviewed and 8 met inclusion criteria for data extraction, qualitative synthesis, and meta-analysis. A summary of the included studies is displayed in Table 2. All included studies were published between 1957 and 2015 [4,9,10,11,24,25,26,27]. Only one study was prospective [26]. The mean modified NOS score for the included studies was 5.25/9.0 in Table 3.

### 3.2. Patient Demographics and Procedures

Of the eight included studies, a total of 97 patients reported an infra-foveal type of femoral head fracture (Pipkin type 1). The average age was 39.3 (31.8~50). Most of the injured patients were male (72.2%) compared to female (27.8%). The mean follow-up time among the studies were 79.7 months. The most common mechanism of injury was motor-vehicle accident (89.7%). Patient demographics are displayed in Table 4.

The procedure details for the infra-foveal type of femoral head fractures are displayed in Table 3. Fragment excision was the most common procedure (n = 45 (46.4%)). Most were documented as small fragment for excision (n = 23/97). Additionally, 27 (27.8 %) of the patients underwent conservative treatment while 25 (25.8%) had open reduction internal fixation.

### 3.3. Post-Traumatic Arthritis

**Figure 2:**
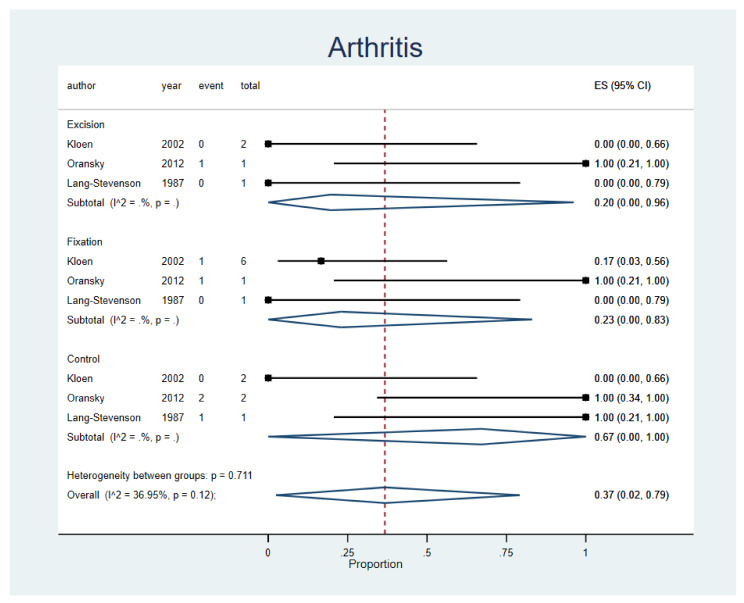
Post-traumatic arthritis. Forest plot indirectly comparing between conservative treatment, fragment excision, and fixation. CI, confidence interval; ES, effect size.

The incidence of post-traumatic arthritis following Pipkin 1 femoral head fractures showed no significant variability either between procedure types (*p* = 0.711) or studies (I^2^ = 36.95%; *p* = 0.12). Conservative treatment alone reported the highest incidence (ES, 67%; 95%CI, 0–100%; n = 3 studies), followed by open reduction internal fixation (ES, 23%; 95% CI, 0–83%; n = 3 studies), and excision (ES, 20%; 95% CI, 0–96%; n = 3 studies). These results are presented in a forest plot in Figure 2. A funnel plot was assessed and displayed in Table S1.

### 3.4. Avascular Necrosis

**Figure 3:**
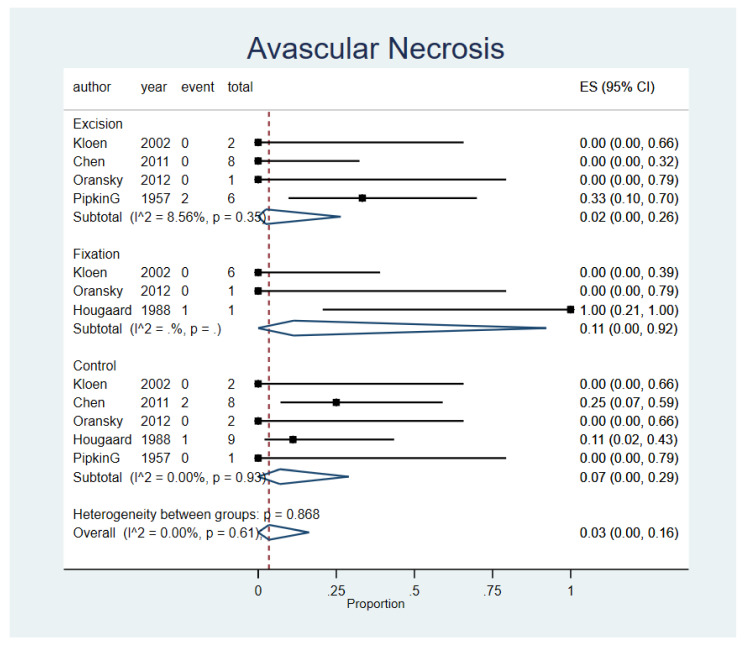
Avascular Necrosis (AVN). Forest plot indirectly comparing between conservative treatment, fragment excision, and fixation. CI, confidence interval; ES, effect size.

The overall rate of Pipkin 1 AVN was 3% (95% CI, 0–16%; n = 5 studies), with no significant variability between either procedures (*p* = 0.868) or studies (I^2^ = 0.00%; *p* = 0.61). Open reduction internal fixation was found to have the highest incidence of AVN (ES, 11%; 95% CI, 0–92%; n = 3 studies), then conservative treatment (ES, 7%; 95% CI,0–29%; n = 5 studies), and excision (ES, 2%; 95% CI,0–26%; n = 4 studies). These results are graphically shown in Figure 3. A funnel plot was assessed and displayed in the Supplementary Materials.

### 3.5. Heterotopic Ossification

**Figure 4:**
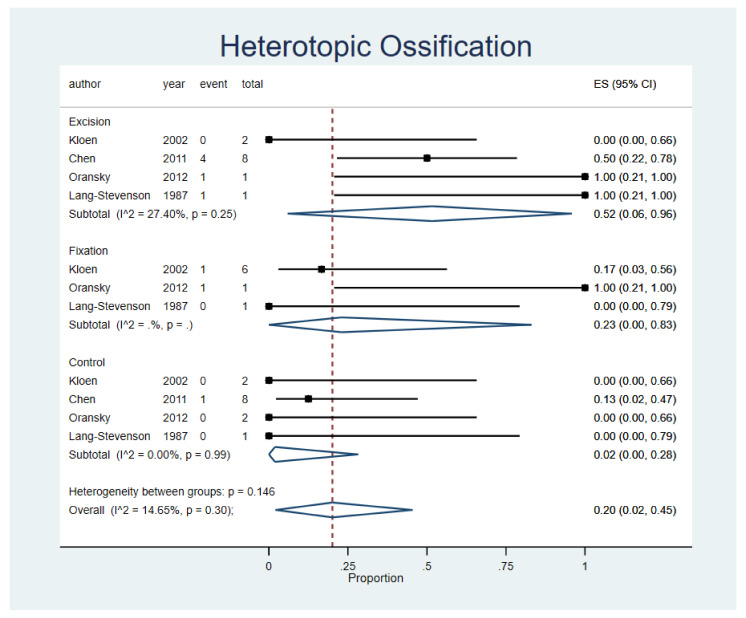
Heterotopic Ossification (HO). Forest plot indirectly comparing between conservative treatment, fragment excision, and fixation. CI, confidence interval; ES, effect size.

The overall HO rate was 20% (95% CI, 2–45%; n = 4 studies) with no significant variability between either procedure groups (*p* = 0.146) or studies (I^2^ = 14.65%; *p* = 0.30). The highest rate was observed in the excision group (ES, 52%; 95% CI, 6–96%; n = 4 studies), then fixation group (ES, 23%; 95% CI,0–83%; n = 3 studies) followed by the conservative group (ES, 2%; 95% CI, 0–28%; n = 4 studies) These findings are presented in Figure 4. A funnel plot was assessed and displayed in the Supplementary Materials.

### 3.6. Thompson Epstein Score and Merle d’ Aubigne and Postel Score

**Figure 5:**
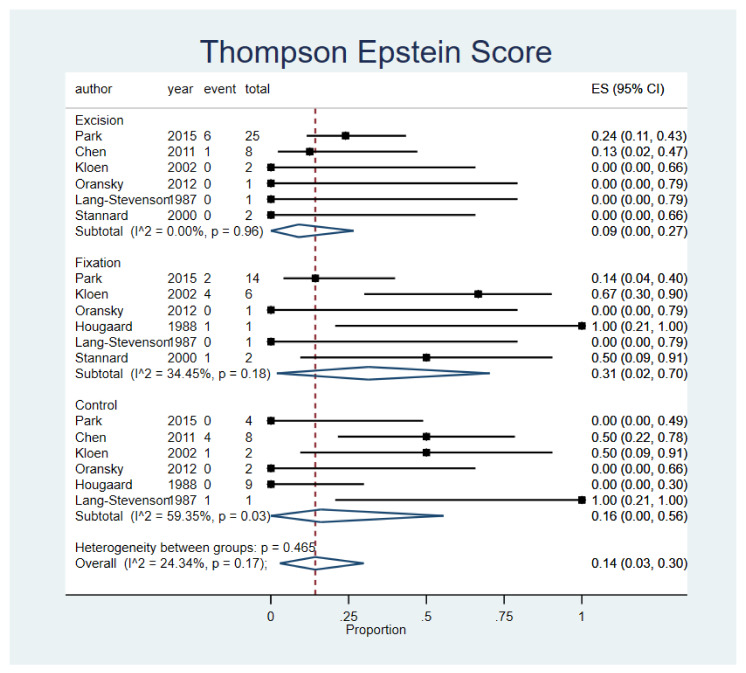
Thompson Epstein Score. Forest plot indirectly comparing between conservative treatment, fragment excision, and fixation. CI, confidence interval; ES, effect size.

**Figure 6:**
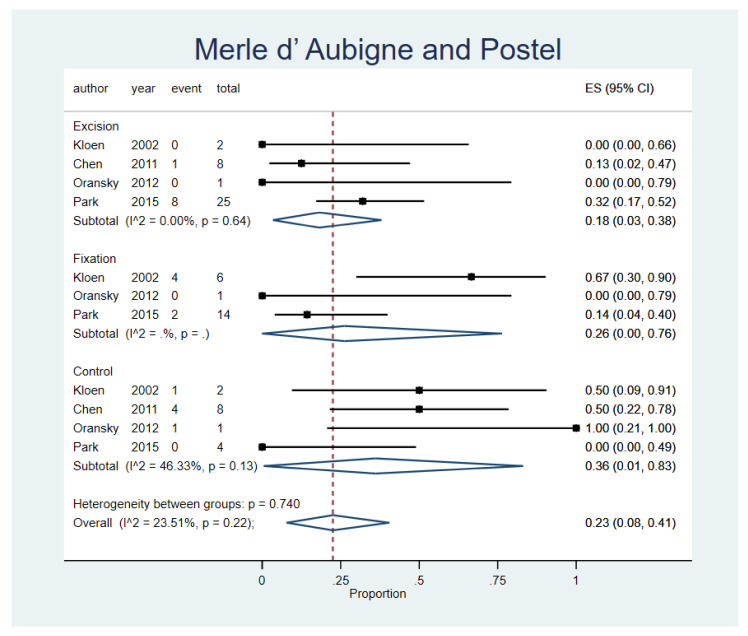
Merle d’ Aubigne and Postel Score. Forest plot indirectly comparing between conservative treatment, fragment excision, and fixation. CI, confidence interval; ES, effect size.

Patients who underwent fragment excision reported the lowest rate of poor to worse outcomes in both Thompson Epstein scores and Merle d’ Aubigne and Postel Score (ES, 9% 95% CI, 0–27%; n = 6 studies, and ES, 18% 95% CI, 3–38%; n = 4 studies); however, no significant variability between procedures (*p* = 0.96) or between studies was observed (*p* = 0.64). Results are presented in Figure 5 and Figure 6. A funnel plot was assessed and displayed in the Supplementary Materials.

## 4. Discussion

The aim of this study was to report a systematic review and meta-analysis of the treatment outcomes following Pipkin type 1 femoral head fractures. Our findings suggest that excision has the best functional outcomes in both functional scores: Thompson Epstein Score (poor to worse ES, 9%; 95% CI, 0% to 27%) and Merle d’ Aubigne and Postel Score (poor to worse ES, 18%; 95% CI, 3% to 38%); ORIF had the highest AVN rate (ES, 11%; 95% CI, 0–92%); conservative treatment had the highest arthritis rate (ES, 67%; 95% CI: 0–100%) and lowest HO rate (ES, 2%; 95% CI, 0–28%).

With regards to mechanism of injury, femoral head fractures typically occur following motor vehicle injury and associated dashboard injuries. The literature has reported that the most common cause of trauma mechanism is MVAs for femoral head fracture, which is reported to be between 84–92% [4,5,6,33,34,35,36]. In this study, 89.7% of patients sustained a femoral head fracture following a MVA while 5.2% and 1.0% of patients fell from either a height or direct impact from falling debris, respectively. Early detection and closed reduction are critical in the initial patient management, especially among poly-trauma patients as femoral head fracture can be undetected.

Type I Pipkin femoral head fractures are inferior to the fovea capitis femoris, the non-weight bearing surface of the femoral head. Appropriate treatment options for Pipkin Type I femoral head fractures must consider joint reduction, hip stability, and congruent joint line. The presence of intra-articular incarcerated fragments impedes a congruent joint reduction [24,25,37]. Chakraborti et al. recommended that conservative management should always be considered first [38]. Historically, femoral head fractures were treated conservatively with prolonged bed rest, in-line traction, and closed reduction. Non-operative management in Pipkin Type I femoral head fractures can be considered if closed reduction achieves <1–2mm displacement and an anatomic congruent hip joint without evidence of fragment interposition. Henle et al. reported that only 1 of 12 patients was in anatomic position following closed reduction [2]. In our analysis, conservative treatment with closed reduction alone for the dislocated hip joint increased the rate of posttraumatic arthritis as well as leading to poor Merle’ d Augine and Postel outcome scores. Supporting our findings, Chen et al. conducted a randomized control trial to assess functional outcomes of 16 Type I Pipkin fractures who either receive closed reduction or closed reduction with fragment excision [26]. Thompson and Epstein and Merle d’Aubigne and Postel scores were both worse for conservative treatment with closed reduction alone (*p*  =  0.032).

Holmes et al. conducted a biomechanical cadaveric study indicating that excision of a small part (<1/3) of the non-weight-bearing surface does not lead to adverse long-term clinical implications [39]. Contrastingly, the literature has reported that retained intracapsular fragments contribute to synovial joint degeneration, chondrocyte apoptosis, and soft tissue destruction [40]. Our study also agreed that conservative treatment alone had the largest incidence of post-traumatic arthritis, compared to fragment excision and open reduction internal fixation.

Fragment size is also critical to predict the prognosis such as the rates of posttraumatic arthritis, AVN, and HO. To further guide appropriate treatment of different fragment size in Pipkin Type I, Yoon et al. modified this classification to: (a) small fragment or several fragments require fragment excision; (b) large fragment requires fragment anatomical reduction [34]. Unfortunately, most studies did not show a consistent inclusion criteria of fragment size. Therefore, the role of different fragment size which impact on treatment decision was difficult to draw conclusions from the literature [34].

Fragment excision as well as open reduction internal fixation are viable surgical options for isolated femoral head fracture, yet results remain inconclusive. Pape et al. reported that 75% of patients reported satisfactory outcomes following closed anatomic reduction alone of Pipkin Type I femoral head fractures as compared to 64% and 50% who either underwent ORIF or fragment excision, respectively. Contrastingly, Giannoudis et al. reported that among 71 Pipkin Type I femoral head fractures, patients who underwent fractured fragment excision reported an 86.7% “excellent” or “good” Thomson-Epstein functional outcome scores, yet not significant when compared to ORIF patients (*p* = 0.07). Furthermore, Epstein et al. reported that among 242 posterior-femoral head fracture dislocations, satisfactory results were achieved in 12%, 42%, and 63% of patients that either received closed reduction alone, closed reduction followed by open reduction, and primary open reduction, respectively [39]. In our study, the results indicate that fragment excision had better functional outcomes in both Thompson Epstein and Merle’ d Augine and Postel Scores when compared to conservative treatment alone or ORIF. We suggest a conservative treatment with closed reduction as the first step for these types of injuries. If a noncongruent hip joint remains, excision or ORIF should be considered accordingly to the fragment size. A small fragment may be excised while a large fragment may be treated with ORIF by screw fixation.

### Limitations

This is the first systematic review and meta-analysis highlighting treatment outcomes according to Type I Pipkin fracture treatment options, yet some limitations must be addressed. First, of the studies evaluated, only one was a prospective randomized control trial. Second, when assessing operative interventions, surgical approach was not evaluated. From the current literature, the Kocher Langenbach approach may show the highest incidence of HO while the transgluteal approach may exist with the highest incidence of AVN. Third, even with aggregated data, the overall sample size had limited power to determine statistical differences stratified by treatment type. It should be noted that our analyses included the largest number of Type I Pipkin Femoral head fractures. Given the infrequency and inconsistency of femoral head fracture treatment guidelines, it is challenging to conduct prospective analysis. It should be noted that Pipkin type I fracture can be treated conservatively or operatively only if anatomical reduction of the infrafoveal fragment. However, while a non-congruent joint (joint mice, 180 degree rotated infrafoveal fragment, etc.) is found after closed reduction for the dislocated hip joint, surgical intervention (excision or fixation) should be considered. Most prior published work limits the generalizability of those findings including either case or single institutional reports [13], and various surgical methods. We still need large multicenter randomized control trials to better understand the optimal treatment for Type I Pipkin femoral head fractures.

## 5. Conclusions

This meta-analysis first demonstrated the clinical outcomes of Type I Pipkin Femoral head fractures and how they differ among procedures. A fragment excision may achieve better function; conservative treatment might result in a higher arthritis rate, and ORIFs may have higher avascular necrosis incidence. These findings may assist surgeons in tailoring their decision-making to specific patient profiles. Future multicenter randomized controlled trials are required to validate associations found in this study.

## Figures and Tables

**Table 1:** Modified Newcastle-Ottawa Scale for assessing study quality.

Study	Selection	Comparability	Exposure/Outcome	Total
Pipkin et al. (1957) [11]	2	NA	3	5
Lang-Stevenson et al. (1987) [24]	2	NA	3	5
Hougaard et al. (1988) [9]	2	NA	3	5
Stannard et al. (2000) [10]	2	NA	3	5
Kloen et al. (2002) [25]	2	NA	3	5
Chen et al. (2010) [26]	4	2	3	9
Oransky et al. (2012) [27]	1	NA	3	4
Park et al. (2015) [4]	2	NA	3	5

NA: not applicable.

**Table 2:** Study characteristics.

Study	Study Design	Total Patient Number, n (%)	Pipkin 1 Cases, n (%)
Pipkin et al. (1957) [11]	Retrospective	24 (12)	7 (7.2)
Lang-Stevenson et al. (1987) [24]	Retrospective	7 (3.5)	3 (3.1)
Hougaard et al. (1988) [9]	Retrospective	19 (9.5)	10 (10.3)
Stannard et al. (2000) [10]	Retrospective	22 (11)	4 (4.1)
Kloen et al. (2002) [25]	Retrospective	32 (16)	10 (10.3)
Chen et al. (2010) [26]	Prospective	16 (8)	16 (16.5)
Oransky et al. (2012) [27]	Retrospective	21 (10.5)	4 (4.1)
Park et al. (2015) [4]	Retrospective	59 (29.5)	43 (44.3)
Total		200	97

**Table 3:** Fragment size and Treatment choice.

Treatment Choice	Pipkin 1 Cases (n = 97)
Excision fragment, n (%)	45(46.4)
Large fragment	11
Small fragment +	23
Not classified	11
Open reduction internal fixation, n (%)	25(25.8)
Close reduction, n (%)	27(27.8)

+ Lang-Stevenson et al. recorded one case as 1/5 of head broken off, and we classified this as small.

**Table 4:** Patient demographics.

Characteristics	Pipkin 1 Cases (n = 97)
Age (yrs) *	39.3
Gender, n (%)	
Male	70 (72.2)
Female	27 (27.8)
Follow-up, months	79.7
Mechanism of injury, n (%)	
Motor-vehicle accident	87 (89.7)
Fall Injury	5 (5.2)
Hit by a falling wall	1 (1.0)
Not recorded	4 (4.1)

* yrs: years.

## Data Availability

Not applicable.

## Supplementary Materials

The following supporting information can be downloaded at: https://www.mdpi.com/article/10.3390/life12010071/s1, Table S1: Full search strategy.

## Abbreviations

HO	Heterotopic Ossification
AVN	Avascular Necrosis
CI	Confidence Interval
ES	effect size
RCT	Randomized Controlled Trial
ORIFs	open reduction internal fixations
WA	weighted averages
SDs	standard deviations
